# Impact of Layered Perovskite Oxide La_0.85_Yb_0.15_AlO_3_ on Structure and Transport Properties of Polyetherimide

**DOI:** 10.3390/ijms24010715

**Published:** 2022-12-31

**Authors:** Alexandra Pulyalina, Valeriia Rostovtseva, Ilya Faykov, Natalia Saprykina, Alexandra Golikova, Anna Fedorova, Galina Polotskaya, Alexander Novikov

**Affiliations:** 1 Institute of Chemistry, Saint Petersburg State University, 198504 Saint Petersburg, Russia; 2 Institute of Macromolecular Compounds, Russian Academy of Sciences, 199004 Saint Petersburg, Russia; 3 Research Institute of Chemistry, Peoples’ Friendship University of Russia (RUDN University), 117198 Moscow, Russia

**Keywords:** layered perovskite oxide, polyetherimide, composite, structure, membrane, gas separation, pervaporation

## Abstract

This study aims to improve properties of Ultem^®^ polyetherimide (PEI) by incorporating up to 2 wt% additives of the perovskite oxide La_0.85_Yb_0.15_AlO_3_ (LYA). The structure of dense PEI/LYA films was characterized by X-ray diffraction (XRD) and scanning electron microscopy (SEM) in combination with an analysis of their elemental composition using energy-dispersive spectroscopy (EDS). The PEI/LYA films exhibit a two-layer structure. Contact angle measurements revealed hydrophilization of the membrane surface enriched with the perovskite. The transport properties were tested via gas separation and pervaporation processes. The separation selectivity of He/N_2_ and O_2_/N_2_ gas pairs increased with the growth of the LYA content in the membranes. Pervaporation of a methanol(MeOH)–cyclohexane(CH) mixture was effective due to the high sorption of MeOH in the PEI/LYA membranes. The maximal pervaporation separation index was found for the PEI/LYA(2%) membrane.

## 1. Introduction

Composite materials are in great demand for most of the leading modern industries and advanced technologies. The latter include membrane technologies, which have such advantages as environmental friendliness, low power consumption, compact equipment, and high selectivity [1]. Membranes are produced from a relatively small number of commonly used materials [2]. However, solving advanced challenges and improving separation processes require the adjustment of membrane properties [3,4,5]. In these cases, the simplest solution is to create composites by incorporating inorganic components into a polymer matrix [6,7]. Polyheteroarylenes are considered to be promising materials for diffusion membrane processes such as pervaporation and gas separation [8,9,10]. The object of this work is Ultem^®^ polyetherimide (PEI)—a thermoplastic material that has been successfully studied in a number of membrane processes [11,12,13]. In this polymer, aromatic imide units provide rigidity and heat resistance, while mobile groups such as -O- and -C(CH_3_)_2_ impart flexibility to macromolecular chains, ensuring the effectiveness of separation processes [14,15]. The analysis of works devoted to the study of PEI membranes has shown that this material has high selectivity with a low level of permeability. To improve its transport properties, the physical modification of PEI with perovskite-type oxides was carried out in the present study.

The structure of a cubic perovskite (Pm3m) is represented by the general formula ABO_3_, in which BO_6_ octahedron moieties form a three-dimensional network, where B-site cations tend to be occupied by smaller transition metal ions with sixfold oxygen coordination. At the same time, larger rare-earth or alkali metal tends to occupy A-sites, with 12-fold oxygen coordination. Compared to other known oxides, the main advantages of perovskite oxides are their electronic structural versatility and compositional flexibility [16]. Owing to their attractive physical and chemical characteristics—such as electronic conductivity, the oxide ions’ mobility through the crystal lattice, thermal and chemical stability, and superparamagnetic and photocatalytic properties—perovskite oxides are useful for wide applications in catalysis, electrochemical sensing, solid oxide fuel cell cathodes, oxygen separation membranes, and membrane reactors for syngas generation [17,18,19]. Recently, layered perovskite oxides have attracted considerable attention as components of composite materials [18,20,21,22]. The use of the layered perovskite oxide K_2_La_2_Ti_3_O_10_ as a modifier of the Torlon^®^ polyamide-imide membrane led to the formation of a unique structure and the improvement of transport properties in pervaporation of an ethanol–ethyl acetate mixture [23]. It has been shown that a dual-phase membrane including the layered perovskite La_1·5_Sr_0·5_NiO_4±δ_ in a molten carbonate is promising for the capture of CO_2_ from simulated flue gas [22].

In recent years, oxide ceramics adapted by atoms of rare-earth elements have aroused great interest among researchers [24,25]. The presence of 4f-localized electrons in the atoms of rare-earth elements distinguishes the properties of these compounds doped with lanthanide atoms from other doped ceramics. The simpler electronic structure of the ytterbium atom compared to other lanthanides, as along with the stability of the Yb^3+^ state, makes it an attractive dopant for further study as a part of multicomponent systems and composites. The radii of lanthanum and ytterbium atoms are 0.136 nm and 0.118 nm, respectively [26]. Substitution of a portion of lanthanum atoms in La_1−y_R_y_AlO_3_ (R—rare-earth element) with ytterbium atoms should lead to various kinds of distortions in the structure of perovskite, which may affect its properties as a membrane modifier. The stability of the perovskite structure depends on the amount of the doping element. In concentrated samples of La_1−y_R_y_AlO_3_, several crystalline phases can form at a high value of y. In this regard, one of the tasks of this work was to obtain a single-phase sample of La_1−y_Yb_y_AlO_3_ in which 15 mol% aluminum was replaced with ytterbium (y = 0.15).

Among the tasks of separating polar/non-polar liquids, the separation of a methanol(MeOH)–cyclohexane(CH) mixture with a point of heteroazeotropic composition (37.2 wt% methanol and 62.8 wt% cyclohexane at 20 °C) was carried out in this work. Cyclohexane is a part of petroleum products; however, it is impossible to isolate pure cyclohexane from a mixture of hydrocarbons by means of rectification. Therefore, azeotropic distillation with methanol is applied in this case, after which the problem of separating a methanol–cyclohexane mixture remains. The use of cyclohexane for removing water from the reaction mixture during esterification is one of the important applications of cyclohexane. However, cyclohexane forms an azeotropic mixture with methanol, which makes it difficult to purify cyclohexane by conventional methods for its subsequent use [27]. There is some experience in using the pervaporation process to separate a methanol–cyclohexane mixture [28,29,30,31].

The aim of this work was to develop novel composites by including additives of the perovskite oxide La_0.85_ Yb_0.15_ AlO_3_ (LYA) in the Ultem^®^ polyetherimide (PEI) matrix and to study the effects of the LYA additives on the structural, physicochemical, and transport properties of PEI-based membranes. The morphology and physicochemical properties of the obtained membranes were studied by scanning electron microscopy (SEM) and atomic force microscopy (AFM), X-ray diffraction (XRD) analysis, sorption experiments, and contact angle measurements. The transport properties of the PEI-based membranes were tested in pervaporation of methanol–cyclohexane mixtures and in gas separation by measuring He, O_2_, and N_2_ permeability.

## 2. Materials and Methods

### 2.1. Materials

Polyetherimide (PEI) with a density of 1.27 g/cm^3^ was used as a polymer matrix (Sigma-Aldrich Chemie GmbH, Schnelldorf, Germany). N-methyl-2-pyrrolidone (NMP), methanol, and cyclohexane were supplied by Vecton (Saint Petersburg, Russia) and used as received. All of the gases used for the permeation tests were provided by Atmosphere (Saint Petersburg, Russia) at a minimum purity of 99 wt%. Lanthanum and ytterbium oxides (99.9 wt%) were supplied by Vekton (Saint Petersburg, Russia).

**Figure 1:**
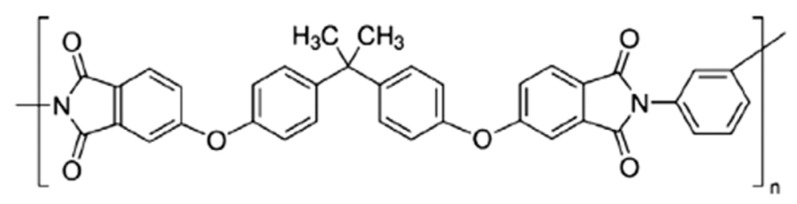
Formula of Ultem^®^ polyetherimide.

The structure of PEI is shown in Figure 1.

### 2.2. Synthesis of Perovskite

A solid solution of La_0.85_Yb_0.15_AlO_3_ (LYA) was obtained by the ceramic method described in [32]. The starting materials were oxides of lanthanum and ytterbium as well as γ-Al_2_O_3_ obtained by thermal decomposition of analytical-grade aluminum nitrate. In order to evaporate the adsorbed water, oxides of lanthanum and ytterbium were calcined at a temperature of 1073 K for 8 h with intermediate homogenization. The phase composition and purity of all of the starting substances were controlled by the X-ray phase analysis. The starting materials were homogenized in stoichiometric amounts in a jasper mortar for 1 h. The resulting powder was tableted using organic glass molds and calcined at a temperature of 1723 K for 60 h. Calcination was carried out until a single-phase sample was obtained, which was controlled by X-ray phase analysis. During sintering, we controlled the constancy of the magnetic susceptibility of the samples. The samples were calcined until the constant magnetic susceptibility was reached to obtain a solid solution with a distribution of atoms in the crystal lattice close to equilibrium.

The content of ytterbium atoms in the solid solutions was determined by the method of inductively coupled plasma atomic emission spectroscopy (ICP-AES Optima 7000 DV, PerkinElmer, Inc., MA, USA). The error did not exceed 5%.

**Figure 2:**
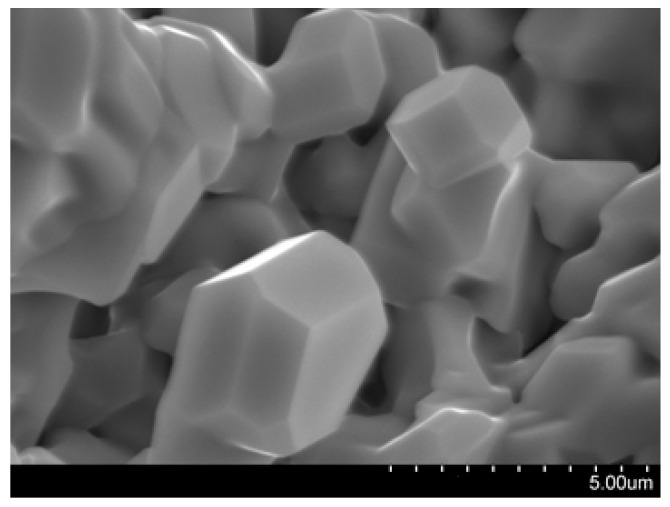
SEM micrograph of La_0.85_Yb_0.25_AlO_3_.

The peculiarities of the sample morphology were studied by the SEM method. The micrograph of the La_0.85_Yb_0.25_AlO_3_ surface (Figure 2) shows cubic particles with a pronounced habitus.

**Figure 3:**
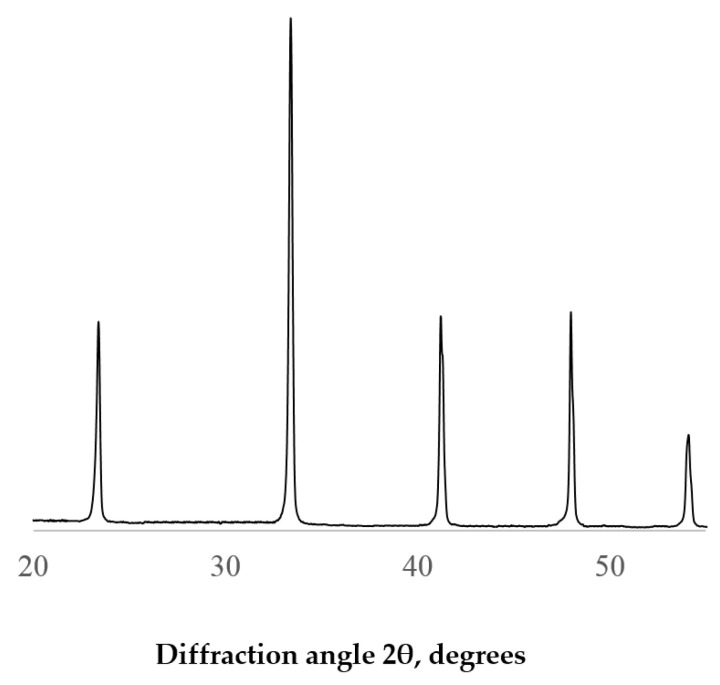
XRD of La_0.85_Yb_0.15_AlO_3_.

**Figure 4:**
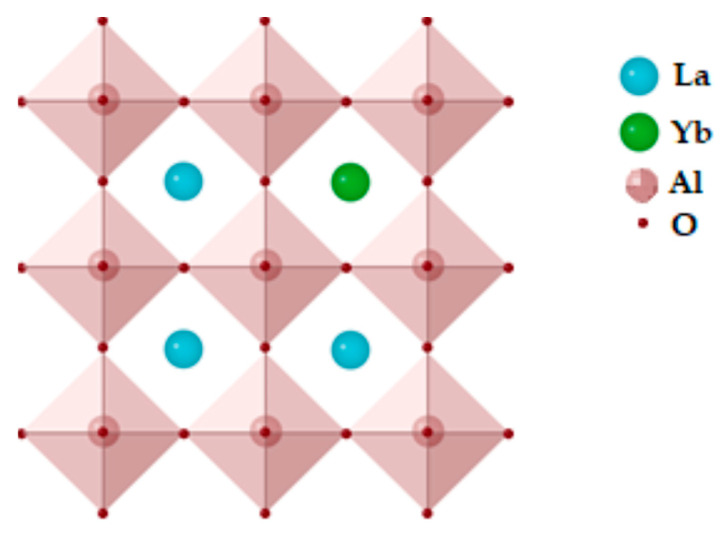
La_0.85_Yb_0.15_AlO_3_ structure.

The X-ray phase analysis of the samples was carried out using a Rigaku MINIFLEX X-ray powder diffractometer (Rigaku Corporation, Tokyo, Japan) with CuKα radiation. The X-ray powder diffraction patterns were identified using the PDF2 database. According to the XRD data (Figure 3), the obtained solid solution was single-phase and had an LYA orthorhombic (Pmcn) perovskite structure (Figure 4). Crystal phases of the starting substances or intermediate products were not detected.

### 2.3. Membrane Preparation

The PEI/LYA composites were prepared from 10 wt% solutions of PEI in NMP by adding LYA perovskite in amounts that provided the required concentrations of the components within the composite. Sonication was used prior to casting to avoid agglomeration and outgassing of the solvent. In the first stage, it was necessary to uniformly disperse the particles in a casting solution via sonication in a BANDELIN HD 2070.2 ultrasonic homogenizer (BANDELIN electronic GmbH & Co. KG, Berlin, Germany) at 40 °C for 60 min. Dense PEI and PEI/LYA membranes were prepared by coating the resulting solution onto a glass plate. The solvent was removed by evaporation at 50 °C for 48 h; the membranes were separated from the glass plate and dried in a vacuum oven at 60 °C to a constant weight. The membrane thickness was 40–50 µm.

### 2.4. Computational Details

The full geometric optimization of all model structures was carried out at the ωB97XD/CEP-121G level of theory using the Gaussian-09 program package [33]. No symmetry restrictions were applied during the geometric optimization procedure. The Hessian matrices were calculated for all optimized model structures to prove the location of correct minima on the potential energy surface (no imaginary frequencies were found in all cases). The thermodynamic parameters were calculated at 298.15 K and 1.00 atm (Tables S1 and S2).

### 2.5. Membrane Characterization

The XRD analysis was performed on a D8 DISCOVER X-ray diffractometer (Bruker, Bremen, Germany) equipped with a CuKα radiation source with a wavelength of 1.54 Å. Scans were conducted with a step size of 0.058, ranging from 5° to 50°.

The surface and cross-section structures of the membranes were studied using a Zeiss SUPRA 55VP scanning electron microscope (Carl Zeiss, Oberkochen, Germany) equipped with In-lens SE and SE2 secondary electron detectors, a secondary electron detector for low-vacuum mode (VPSE), and a four-quadrant backscattered electron detector (AsB). The samples were coated with a 20 nm thick platinum layer using the Quorum 150 cathode sputtering installation (Quorum Technologies Ltd., Lewes, UK) prior to the experiment.

An NT-MDT NTegra Maximus atomic force microscope (NT-MDT Spectrum Instruments, Zelenograd, Russia) with standard silicon cantilevers (stiffness of 15 N⋅m^−1^ in tapping mode) was used to study the topography of the membranes.

The membrane density (*ρ_exp_*) was experimentally measured by the flotation method using a mixture of toluene and carbon tetrachloride to equilibrate the specimens at 20 °C (*ρ*_toluene_ = 0.867 g/cm^3^, *ρ*_CCl4_ = 1.594 g/cm^3^).

The calculated density (*ρ_calc_*) of the PEI-LYA membranes was estimated as follows [34,35,36]:

(1) $$\rho_{calc}=\frac{1}{\frac{1-w_{LYA}}{\rho_{PEI}}+\frac{w_{LYA}}{\rho_{LYA}}}$$

where *ρ_PEI_* and *ρ_LYA_* are the densities of PEI and LYA, respectively, while *w_LYA_* is the weight fraction of LYA in a composite.

The contact angles were measured via the sessile drop technique using a DSA 10 drop shape analyzer (KRÜSS GmbH, Hamburg, Germany) at room temperature and atmospheric pressure, using water and ethylene glycol. Based on the measured values of the contact angles, the critical surface tension (*σ_s_*) and its dispersion $\sigma_{S}^{d}\;$ and polar $\sigma_{S}^{p}$ components were calculated by the Owens–Wendt method [37]:

(2) $$\sigma_{S}=\sigma_{S}^{d}+\sigma_{S}^{p}$$

The liquid sorption was studied by immersing the membrane samples in pure liquids (methanol or cyclohexane) at 20 °C. At certain intervals, the membrane samples were removed from the liquids and weighed on a Mettler Toledo ME204 analytical balance (Mettler Toledo, Columbus, OH, US) with an accuracy of 10^−4^ g. The experiment continued until the equilibrium state was reached. The equilibrium sorption degree (*S*) was calculated by the following equation:

(3) $$S=\frac{m_{s}-m}{m}\times 100\%$$

where *m_s_* is the membrane sample’s weight after reaching the sorption equilibrium, while *m* is the membrane sample’s weight after carrying out a desorption procedure.

To determine the diffusion coefficient (*D*) of a penetrant through a polymer membrane, the kinetic curve was plotted in coordinates *M_t_/M_∞_* = f (*t*^1/2^/*l*), where *M_t_* is the amount of liquid sorbed during time *t*, *M_∞_* is the maximum amount of sorbed liquid, and *l* is the membrane thickness [38]. In this case, the initial stage of diffusion was considered, which corresponds to the values of *M_t_/M_∞_* < 0.4. The diffusion coefficient was calculated from the slope of the linear part of the curve (*tgα*):

(4) $$D=\frac{\pi }{16}\times {\left(tg\alpha \right)}^{2}$$

### 2.6. Pervaporation

Pervaporation experiments on the separation of MeOH and CH mixtures were carried out using a lab-scale apparatus with stirring at 50 °C, as described in our previous work [39]. The downstream pressure was maintained below 10^−2^ mm Hg on the permeate side with an MD 1C vacuum pump (Vacuubrand GMBH, Wertheim, Germany). The permeate was collected in a trap immersed in liquid nitrogen and weighted on a Mettler Toledo ME204 balance (Mettler Toledo, Columbus, OH, US). The feed and permeate compositions were analyzed using a “Chromatec–Crystal 5000.2” gas chromatograph (Chromatec, Yoshkar-Ola, Russia) with a thermal conductivity detector. The experiments were repeated three times, and the average value of the results was considered.

The total flux through a membrane (*J*) was determined based on the amount of a liquid that penetrated through the membrane area per time unit. To compare the transport properties of membranes with a thickness (*l*) ranging from 40 to 50 µm, the normalized total flux (*J_n_*) value was used. *J_n_* is the flux through a membrane with a thickness of 20 µm, calculated as follows:

(5) $$J_{n}=\frac{J\times l}{20}$$

The separation factor (*β_MeOH/CH_*) was defined via the following equation [40]:

(6) $$\beta_{MeOH/CH}=\frac{Y_{MeOH}/Y_{CH}}{X_{MeOH}/X_{CH}}$$

where *Y* and *X* are the weight fractions of the components in the permeate and the feed, respectively.

The pervaporation separation index (*PSI*), which is a parameter generalizing the transport properties of a membrane, was calculated as follows:

(7) $$PSI=J_{n}\times \left(\beta_{MeOH/CH}-1\right)$$

### 2.7. Gas Separation

Gas permeation tests were performed with single gases with a high purity (He, O_2_, N_2_) by the barometric technique, using a laboratory high-vacuum apparatus described elsewhere [41]. At least two films were examined to ensure the reproducibility of the tests. At the beginning of the permeation experiment, the gas under a constant pressure *p* (150 kPa) was brought into the feed part of the permeation cell. The permeability coefficient was determined from the increase in pressure Δ*p* in a calibrated volume *V_p_* of the product part of the cell per the time interval Δ*t* during steady-state permeation. The gas permeability coefficient (*P*) was estimated using the following equation:

(8) $$P=\frac{\Delta p_{p}}{\Delta t}\cdot \frac{V_{p}\cdot l}{S\cdot p}\cdot \frac{1}{RT}$$

where *l* is the membrane’s thickness, *S* is its area, *T* is the absolute temperature, and *R* is the gas constant. All of the measurements were carried out at 30 °C. The permeability coefficient *P* was expressed in Barrers (1 Barrer = 10^−10^ cm^3^ (STP)cm/(cm^2^ s cmHg)).

The ideal selectivity (*α_i/j_*) for gas *i* with respect to gas *j* was calculated according to the following equation:

(9) $$\alpha_{i/j}=\frac{P_{i}}{P_{j}}$$

## 3. Results and Discussion

### 3.1. Characterization of PEI/LYA Membranes

Dense films based on PEI modified with the La_0.85_ Yb_0.15_ AlO_3_ perovskite-like oxide were prepared by the solution casting method. In our previous work [23], composite membranes containing up to 3 wt% perovskite-type layered oxide K_2_La_2_Ti_3_O_10_ in the Torlon^®^ PAI matrix were studied; it was found that only the inclusion of 1 and 2 wt% perovskite in the polymer membrane enabled the preparation of defect-free membranes. The same effect occurred when preparing PEI/LYA membranes in the present study. Therefore, the data on the samples containing 0, 1, and 2 wt% LYA in the PEI matrix are discussed here. It is well established that the inclusion of inorganic particles in a polymer matrix leads to the formation of either a more compact or looser structure of the polymer films. To elucidate the nature of the structural changes in the PEI films with different LYA contents, the membrane density was determined using two methods: theoretical calculation according to Equation (1), and experimental measurements by the flotation method. The comparative data obtained experimentally and by calculation are presented in Table 1.

The calculated density (*ρ_calc_*) of the films increased upon the inclusion of LYA in the composites and an increase in its content. This fact may be related to the much higher density of perovskite (4.0 g/cm^3^) compared to that of pure PEI (1.267 g/cm^3^). The experimental density (*ρ_exp_*) also increased with the increase in the LYA content. However, for each of the PEI/LYA composites, the value of *ρ_exp_* was lower than that of *ρ_calc_*.

**Figure 5:**
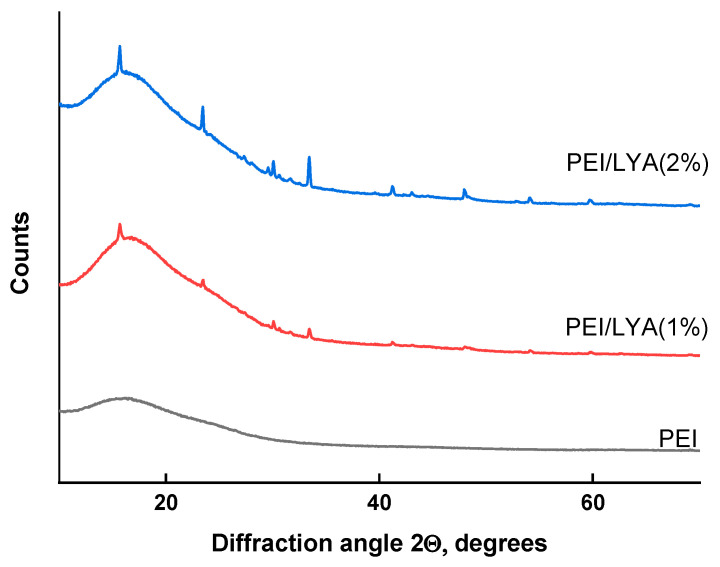
XRD patterns of pure PEI and PEI/LYA membranes.

The structural features of the new composite membranes were evaluated using the XRD analysis. Figure 5 shows the X-ray diffraction patterns of the PEI/LYA composite films compared to those of PEI. The pure PEI film exhibited an amorphous character, and a single broad peak appeared on the X-ray diffraction pattern at about 17°, corresponding to a d-space value of 5.21 Å. The spectra of the PEI/LYA composites were different from those of PEI. In the case of the composites, the broad peak characteristic of PEI was retained. However, sharp peaks were also observed at 2θ = 15.78, 23.56, 30.09, 33.47, and 41.16, characteristic of the perovskite-type oxide (see, Figure 3). The intensity of these peaks increased with the increase in the LYA loading, indicating the possible aggregation of the filler particles.

**Figure 6:**
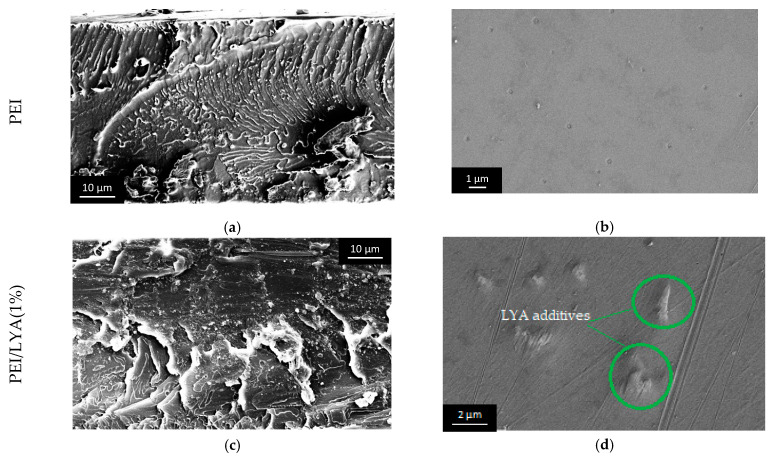

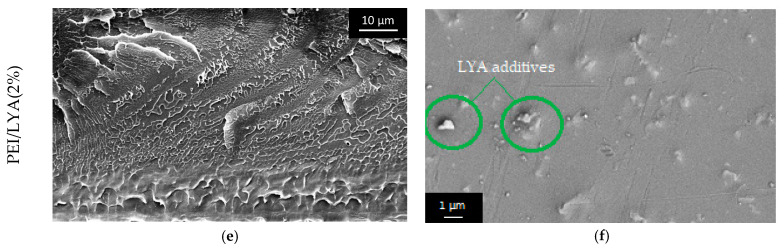
SEM micrographs of the (**a**,**c**,**e**) cross-section (×3000) and (**b**,**d**,**f**) bottom surface (×20,000) of the PEI/LYA membranes.

The peculiarities of the membrane morphology were studied by SEM. Figure 6 shows the micrographs of the cross-section and bottom surface of the PEI-based membranes with varying filler contents. It can be seen that the PEI/LYA membranes exhibit a complex morphology; they are structured and comprise amorphous and crystalline phases. The inclusion of LYA in the PEI matrix changes the structure of the cross-section and bottom surface of the membranes. Large LYA particles with a high density descend to the bottom of the PEI/LYA film during a prolonged time of NMP evaporation from a polymer solution. Figure 6a–e show the cross-sections of the membranes, with a characteristic brittle fracture structure. The micrographs show individual particles and agglomerates (bright dots) of perovskite, especially at the edge near the bottom of the membranes. The micrographs of the bottom surfaces of the membranes (Figure 6b–f) also show particles protruding from the matrix and perovskite agglomerates. Noticeably, more irregularities are observed in the PEI/LYA(2%) membrane compared to PEI/LYA(1%), while the bottom surface of the PEI membrane is absolutely smooth.

**Figure 7:**
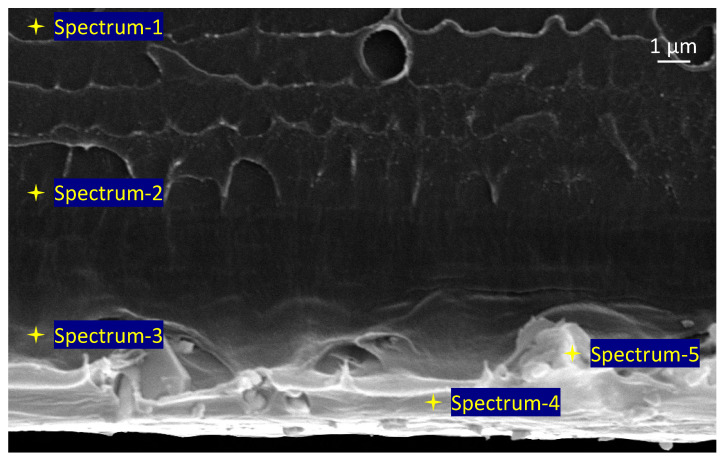
SEM micrographs of the PEI/LYA(2%) membrane, where the positions of spectral points 1–5 are indicated.

The elemental composition of the membranes was studied by energy-dispersive spectroscopy. It was observed that the membranes made of the PEI/LYA composites exhibited different compositions over their cross-section. Table 2 shows the data on the EDS elemental analysis of the PEI/LYA(2%) membrane at points 1–5 of the cross-section. Figure 7 shows the cross-section of the PEI/LYA(2%) membrane, where the positions of the spectral points 1–5 are indicated.

For the PEI/LYA(2%) membrane, the EDS of the top surface contains elements (C, N, O) that constitute the chemical formula of PEI. The composition of the bottom surface, in addition to C, N, and O reduced in quantity, contains the elements Al, La, and Yb, which are also contained in the perovskite formula. Thus, the data shown in Table 2 and Figure 7 indicate an irregular distribution of LYA in the PEI matrix over the depth of the membrane. Spectrum 1 (top) does not contain LYA, which appears in Spectrum 2 and Spectrum 3, while Spectrum 4 and Spectrum 5 (bottom) contain the maximal amount of the La_0.85_Yb_0.15_AlO_3_ elements. The formation of such a structure is due to a significant difference in the density of the membrane components: the PEI density is ~1.27 g/cm^3^, while the LYA density is ~4.0 g/cm^3^. When preparing a casting solution using ultrasound, a homogeneous transparent solution of PEI/LYA(2%) was formed. However, after casting the latter on a glass plate, some LYA particles moved to the bottom under the gravitational forces during the evaporation of NMP at 50 °C for 3–4 h.

**Figure 8:**
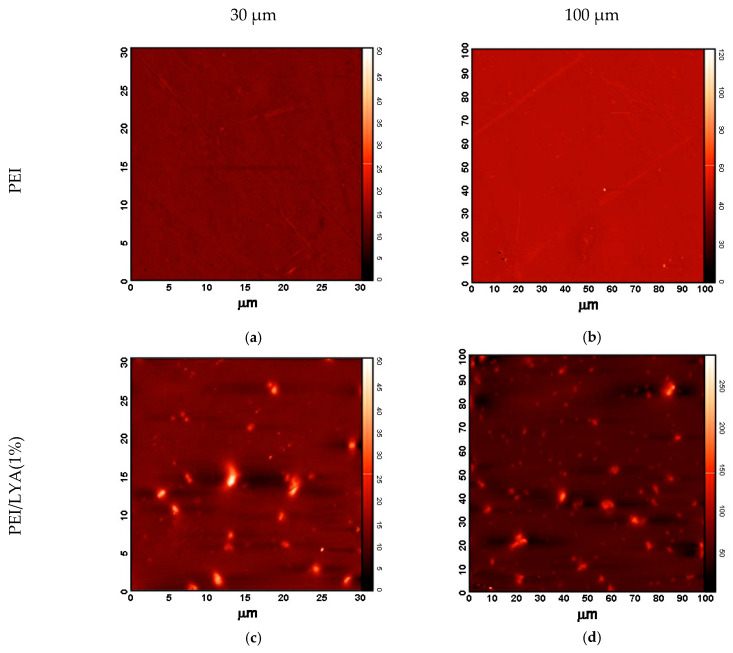

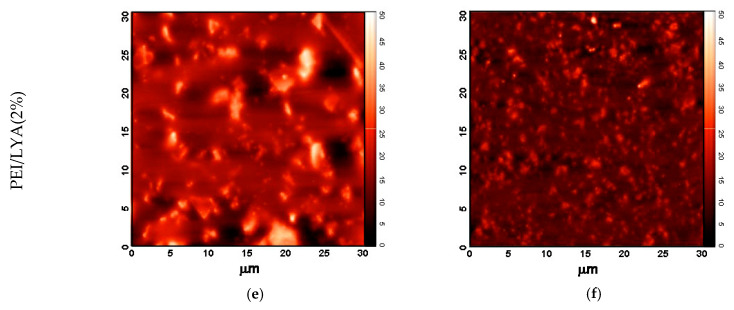
AFM images of the (**a**,**b**) PEI and (**c**–**f**) PEI/LYA membranes.

The surface structure was additionally investigated by AFM to evaluate the surface roughness of the membranes. The AFM data confirmed the conclusion based on the results of SEM with respect to the agglomeration of particles on the bottom of the membrane and the formation of an asymmetric structure. The top surfaces of all of the membranes were actually similar. As shown in Figure 8a,b and Table 3, the membranes containing no modifier had a lower roughness.

The membranes containing the LYA perovskite additives (Figure 8c–f) show a much higher average surface roughness. However, the uniform distribution of particles over the entire surface and the absence of obvious defects should be noted. The membrane with 2% LYA has the maximum surface roughness (the average roughness (Ra) is 116 Å and the root-mean-square surface roughness (Rq) is 165.4 Å), indicating the largest number of particles agglomerated on the membrane surface. An increase in the surface roughness provides a larger effective contact area with the feed components, which is one of the factors that facilitate sorption and faster penetration of substances [42].

The nature of the changes in the surface properties of the PEI membranes modified with perovskite was estimated using the data from measuring the contact angles of water and ethylene glycol on the surface of the PEI/LYA membranes.

**Figure 9:**
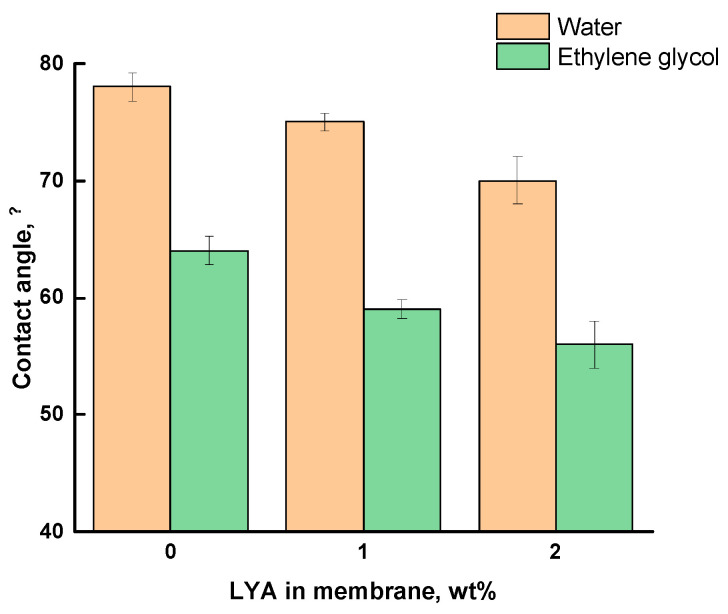
Contact angles of water and ethylene glycol vs. the LYA content in the PEI/LYA membrane.

The measured contact angles of water and ethylene glycol on the surface of the PEI/LYA(0–2 wt%) membranes are summarized in Figure 9. The water contact angle (θ_water_) of pure PEI is equal to 78°, which confirms its moderate hydrophilicity. The latter decreases with the increase in the LYA content in the composite. The ethylene glycol contact angle (θ_EG_) on the membrane surfaces has the same tendency to decrease.

The data on the contact angles were used to calculate the critical surface tension (σ_s_) of the membranes under the study, including the contribution of the polar and dispersion components. Table 4 shows an increase in the polar component of the surface tension for the membranes modified with LYA, which indicates hydrophilization of the membrane’s surface. At the same time, the inclusion of the inorganic modifier LYA in the PEI membrane leads to a decrease in the dispersion component. Since the dispersion component of the surface tension characterizes the packing density of the surface layer segments, it can be concluded that the degree of macromolecular packing changes in the LYA-modified membranes.

### 3.2. Transport Properties

Two membrane methods—pervaporation and gas separation—were selected to estimate the transport properties of the PEI/LYA composites. These processes differ only in the phase state of the feed mixture. In both processes, the transport of molecules through a membrane is carried out according to the “solution—diffusion” mechanism, which is described by the equation *P* = *S·D*, where *P* is the permeability coefficient, *S* is the solubility coefficient, and *D* is the diffusion coefficient.

During gas separation, permeability through a membrane is mainly determined by the diffusion coefficient, which depends on the size of the penetrating molecules, as well as on the size and distribution of the free volume in the polymer membrane. On the other hand, the permeability of liquid molecules during pervaporation is mainly determined by the solubility of the penetrating molecules in the polymer membrane; thus, it depends on the sorption activity of the polymer.

#### 3.2.1. Pervaporation of a Methanol–Cyclohexane Mixture

One of the urgent tasks of pervaporation is the separation of liquids with different polarity, such as a methanol–cyclohexane mixture [43]. The separation effectiveness depends on the selective sorption of molecules in the polymer membrane. Therefore, sorption experiments were carried out for the PEI/LYA(0, 1, or 2%) membranes in individual liquids: methanol and cyclohexane.

It was found that all of the membranes based on PEI were inert with respect to cyclohexane. Since cyclohexane is a nonpolar molecule that does not significantly interact with the hydrophilic polymer, its solubility in the polymer matrix is immeasurably low. Table 5 shows the data on the sorption degree and diffusion coefficients of methanol in the membranes. The degree of methanol sorption increases when LYA is included in the polymer matrix. The LYA additives increase the hydrophilicity of the membranes, with positive effects on the sorption activity of the PEI/LYA membranes. As shown from Table 5, the diffusion coefficients of methanol increase as a result of membrane modification with LYA.

The affinity of methanol towards the PEI matrix was also confirmed by the results of quantum chemical calculations. Interactions of methanol and cyclohexane with PEI were considered as hypothetical supramolecular association processes.

**Figure 10:**
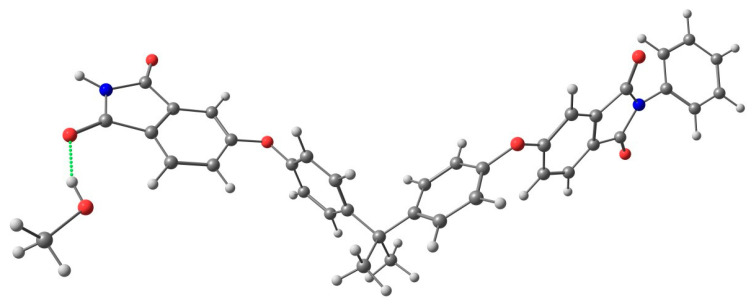
Scheme of coordination of methanol with PEI.

The results of quantum chemical calculations (Tables S1 and S2) reveal that supramolecular association of cyclohexane with PEI is thermodynamically unfavorable (viz. 1.6 kcal/mol in terms of Gibbs free energy of the reaction), whereas the formation of the PEI—MeOH adduct (Figure 10) occurs with negative Gibbs free energy of the reaction (viz. −1.0 kcal/mol).

**Figure 11:**
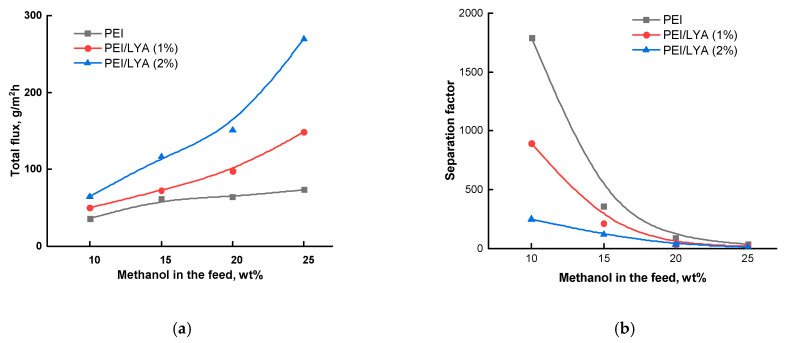
Dependence of (**a**) the total flux and (**b**) the separation factor on the methanol concentration in the feed for pervaporation of the methanol–cyclohexane mixture using the PEI/LYA(0–2%) membranes at 20 °C.

The PEI/LYA(0, 1, or 2%) membranes were studied in the pervaporation of methanol–cyclohexane mixtures containing up to 25 wt% methanol. All of the membranes were predominantly permeable to methanol. Figure 11a,b show the dependencies of the total flux and the separation factor on the methanol concentration in the feed, respectively. With an increase in the methanol concentration in the feed, the total flux through all of the membranes increases, but the separation factor decreases. Figure 11a shows that the inclusion of LYA in the membrane and an increase in its content up to 2 wt% lead to an increase in the total flux; this fact is consistent with an increase in the diffusion coefficient of methanol (Table 5).

Figure 11b shows that the separation factor decreases with the addition of LYA and an increase in its content in the membrane up to 2 wt%. This result can be explained by an increase in the sorption degree of methanol in the PEI/LYA composites, which leads to an increase in the size of the transport channels and facilitates the permeation of larger cyclohexane molecules; the separation factor decreases in this case.

**Figure 12:**
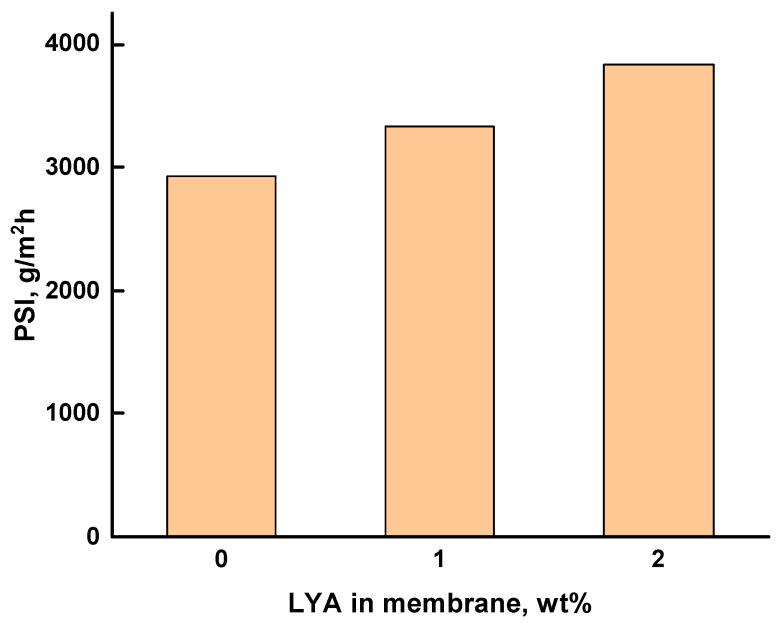
Pervaporation separation index (*PSI*) vs. the LYA content in the membrane during pervaporation of the methanol–cyclohexane mixture (20 wt%).

The effectiveness of the developed membranes was evaluated using the pervaporation separation index (*PSI*), which includes the values of both the total flux and the separation factor for the PEI, PEI/LYA(1%), and PEI/LYA(2%) membranes during pervaporation of the methanol–cyclohexane mixture containing 20 wt% methanol (Figure 12). The best transport properties were found for the membrane containing 2 wt% LYA.

Thus, the use of layered perovskite oxide as a modifier of a polymer membrane is promising for increasing the effectiveness of the process.

#### 3.2.2. Gas Separation

The single-gas permeability of He, O_2_, and N_2_ was determined for the membranes based on PEI and composites containing 1 and 2 wt% LYA. Possible interactions of gas molecules (N_2_, O_2_, He) with PEI were considered as hypothetical supramolecular association processes when performing quantum chemical calculations (Tables S1 and S2). The Gibbs free energies for the formation of the PEI—N_2_ and PEI—He adducts were almost the same (4.4 and 4.5 kcal/mol, respectively), whereas this thermodynamic quantity for the formation of the PEI—O_2_ adduct was markedly different (5.4 kcal/mol).

**Figure 13:**
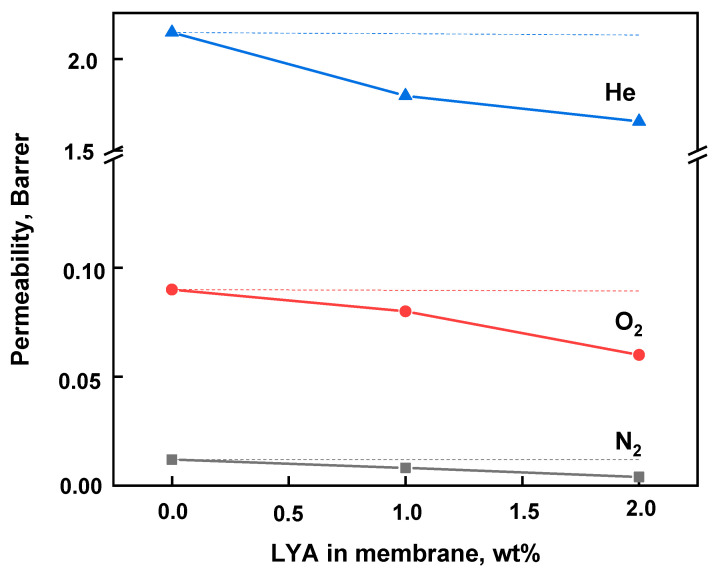
Dependence of gas permeability on the LYA content in the membrane at 20 °C. Dotted lines represent the values of gas permeability calculated according to the Maxwell model [44].

Figure 13 shows the dependence of gas permeability on the LYA content in the membrane. The permeability coefficients for all of the tested gases decreased with an increase in the LYA content in the membranes. Generally, the gas transport properties are determined by the diffusion coefficient, which substantially depends on the free volume of a polymer. Modification of PEI with LYA leads to an increase in the density and a decrease in the free volume, which may contribute to a decrease in the gas permeability of the PEI/LYA membranes.

To evaluate the effects of the LYA modifier on the membrane transport properties, the calculated values of PEI/LYA(0, 1, or 2%) permeability were obtained (shown as dotted lines in Figure 13) using the Maxwell model [45]. Based on the obtained data, it was found that the gas permeability values calculated according to the Maxwell model were much higher than the corresponding experimental values. This probably means that a non-permeable region formed around the inorganic modifier, leading to a decrease in the gas permeability. This effect was most likely due to additional interactions by coordination between the modifier and the polymer through non-covalent forces.

An important factor determining the gas diffusion through a membrane is the effective kinetic diameter of the gas molecules: He (0.178 nm), O_2_ (0.289 nm), and N_2_ (0.304 nm). Thus, the level of permeability decreases in the order He > O_2_> N_2_.

**Figure 14:**
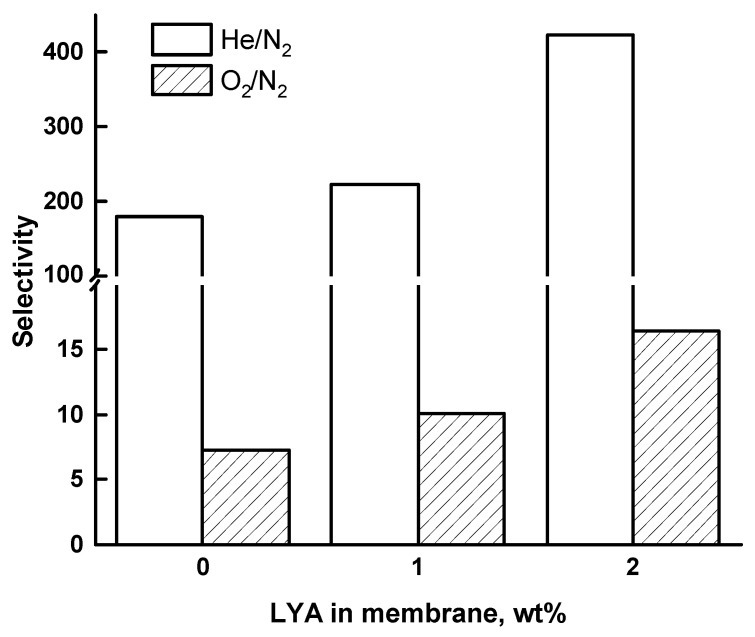
Dependence of ideal selectivity on the LYA content in the membrane for He/N_2_ and O_2_/N_2_ at 20 °C.

Figure 14 shows the dependence of the selectivity on the perovskite content in the PEI/LYA membranes. The greater difference in the permeability of He and N_2_ leads to higher values of He/N_2_ selectivity than that of O_2_/N_2_. It was found that the ideal selectivity in the separation of He/N_2_ and O_2_/N_2_ gas pairs increased with the increase in the LYA content in the membranes.

**Figure 15:**
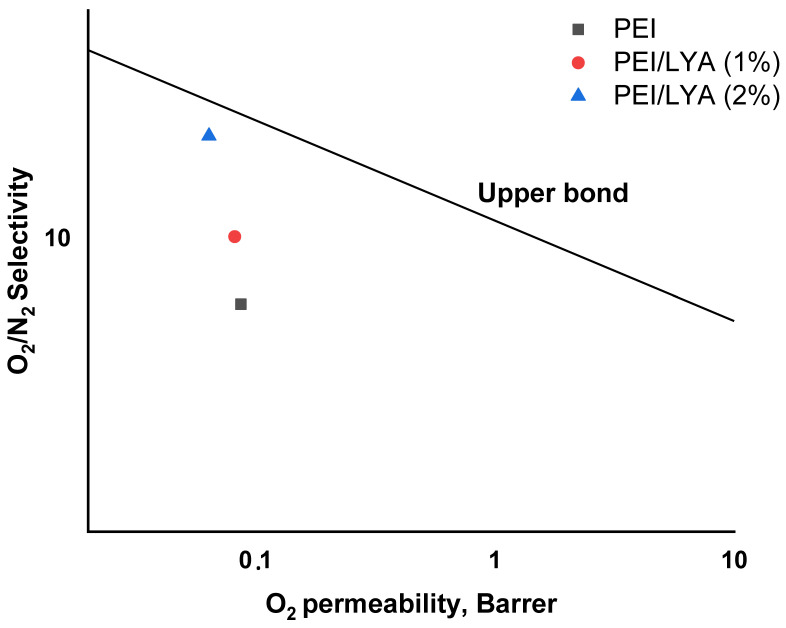
Permselectivity vs. permeability diagram for O_2_/N_2_ gas pairs. The upper bound line was taken from the Robeson diagram [45].

The gas transport properties of the PEI/LYA membranes were compared with those of the membranes shown in the Robeson diagram, wherein the data on gas permeability and selectivity for a number of the studied polymers had been analyzed and the position of the upper boundary had been established previously [45]. The O_2_ permeability and O_2_/N_2_ selectivity for the PEI/LYA(0, 1, and 2%) membranes were plotted on the Robeson diagram with the upper bound line of 2008 (Figure 15). The position of the PEI membrane on the diagram was improved towards higher O_2_/N_2_ selectivity due to the inclusion of LYA in the PEI matrix. This indicates a promising approach for modifying the membrane with La_0.85_ Yb_0.15_ AlO_3_ additives.

## 4. Conclusions

In this research, novel composites were obtained by incorporating 1 and 2 wt% additions of the La_0.85_ Yb_0.15_ AlO_3_ layered perovskite oxide into the Ultem^®^ polyetherimide matrix. The composites were used to form dense membranes. The nature of the structural changes in the PEI membranes was evaluated based on the changes in the membrane density, as well as using the XRD analysis and SEM in combination with the EDS analysis of the elemental composition. It was found that the PEI/LYA membranes have a two-layer structure containing a thin layer highly enriched in LYA (~5 µm), which is organically bound to the matrix (~30 µm). A decrease in the contact angles for water and ethylene glycol on the membrane surface enriched with LYA indicated the hydrophilization of the PEI/LYA membranes.

The transport properties of the PEI/LYA composites during the pervaporation of methanol–cyclohexane mixtures were determined based on the high sorption of methanol. Upon the addition of LYA, the total flux increased and the separation factor decreased; these results were associated with an increased sorption degree of methanol in the PEI/LYA membranes. The maximal pervaporation separation index was found for the PEI/LYA(2%) membrane.

The gas transport properties were determined by measuring the single-gas permeability of He, O_2_, and N_2_ through the PEI/LYA membranes. The permeability coefficients decreased for all of the tested gases with an increase in the LYA content in the membrane. This fact appears to have been determined by an increase in the density and a decrease in the free volume of the composites. The ideal selectivity in the separation of He/N_2_ and O_2_/N_2_ gas pairs increased with the increase in the LYA content in the membranes.

Thus, it was found that layered perovskite oxides as modifiers of polymer membranes are promising for increasing the effectiveness of the gas separation and pervaporation processes, opening up new opportunities for studying such composite materials as high-performance membranes.

## Figures and Tables

**Table 1:** Density of the membranes.

Membrane	*ρ_calc_*, g/cm^3^	*ρ_exp_*, g/cm^3^
PEI	-	1.267
PEI/LYA(1%)	1.276	1.273
PEI/LYA(2%)	1.285	1.278

**Table 2:** EDS elemental analysis of the PEI/LYA(2%) membrane’s cross-section.

Spectrum	C	N	O	Al	La	Yb	Total
	wt%	wt%	wt%	wt%	wt%	wt%	wt%
Spectrum 1 (top)	70.33	9.28	20.29	0.00	0.10	0.00	100.00
Spectrum 2	69.83	8.71	20.64	0.00	0.51	0.31	100.00
Spectrum 3	62.98	8.89	20.96	0.39	3.86	2.92	100.00
Spectrum 4 (Bottom)	57.96	5.23	14.61	2.08	18.80	1.32	100.00
Spectrum 5 (Bottom)	36.13	2.06	19.35	2.51	17.56	22.39	100.00

**Table 3:** The surface roughness of the membranes.

Membrane	Ra, Å	Rq, Å
PEI	6.49 ± 1.2	11.18 ± 1.4
PEI/LYA(1%)	18.97 ± 4.1	41.26 ± 6.7
PEI/LYA(2%)	116 ± 3.9	165.4 ± 19.5

**Table 4:** Data on surface tension, 20 °C.

Membrane	Surface Tension, mJ/m^2^		
	*σ^p^_s_*	*σ^d^_s_*	*σ_s_*
PEI	16.6	10.0	26.6
PEI/LYA(1%)	17.3	11.8	29.1
PEI/LYA(2%)	22.4	10.2	32.6

**Table 5:** The sorption degree and diffusion coefficient of methanol for the PEI/LYA membranes.

Membrane	Sorption Degree, %	Diffusion Coefficient, m^2^/s·10^12^
PEI	14.0	1.7
PEI/LYA(1%)	15.8	1.8
PEI/LYA(2%)	17.6	2.1

## Data Availability

Not applicable.

## Supplementary Materials

The following supporting information can be downloaded at: https://www.mdpi.com/article/10.3390/ijms24010715/s1, Table S1: Calculated enthalpies, entropies, and Gibbs free energies for optimized equilibrium model structures; Table S2: Calculated values of Gibbs free energies of reaction for various hypothetical supramolecular association processes.
