# Supplementary material for: Impact of Layered Perovskite Oxide La0.85Yb0.15AlO3 on Structure and Transport Properties of Polyetherimide
Source: Int J Mol Sci. 2022 Dec 31;24(1):715. doi: 10.3390/ijms24010715 (PMC9821444; doi:10.3390/ijms24010715)
Supplement: Supplementary file 1 [file ijms-24-00715-s001.zip › ijms-2094141-supplementary.pdf]

Supplementary Materials

Table S1. Calculated enthalpies, entropies, and Gibbs free energies (in Hartree) for optimized equilibrium model structures (H, S, and G, respectively).

Model Structure	H	S	G
Cyclohexane	-40.953141	74.604	-40.988588
MeOH	-23.932299	56.951	-23.959359
N_2	-19.763962	45.875	-19.785759
O_2	-31.778169	49.153	-31.801523
He	-2.905283	30.125	-2.919596
Ultem	-339.482655	232.263	-339.593011
Ultem–cyclohexane	-380.450769	269.864	-380.578990
Ultem–MeOH	-363.430548	259.779	-363.553977
Ultem–N_2	-359.251122	253.892	-359.371754
Ultem–O_2	-371.264797	255.109	-371.386008
Ultem–He	-342.387718	247.809	-342.505460

Table S2. Calculated values of Gibbs free energies of reaction (ΔG, in kcal/mol) for various hypothetical supramolecular association processes.

Supramolecular Association Process	ΔG
Cyclohexane + Ultem → Ultem–cyclohexane	1.6
MeOH + Ultem → Ultem–MeOH	–1.0
N_2 + Ultem → Ultem–N_2	4.4
O_2 + Ultem → Ultem–O_2	5.4
He + Ultem → Ultem–He	4.5
